# Descriptors of living alone for elders: based on Turkey national data

**DOI:** 10.1186/s12877-021-02706-9

**Published:** 2022-01-10

**Authors:** Filiz Adana, Seyfi Durmaz, Safiye Özvurmaz, Ceren Varer Akpınar, Duygu Yeşilfidan

**Affiliations:** 1 Faculty of Nursing, Department of Public Health Nursing, Aydın Adnan Menderes University, Aydın, Turkey; 2 Faculty of Medicine, Department of Public Health, Ege University, İzmir, Turkey; 3 Faculty of Medicine, Department of Public Health, Giresun University, Giresun, Turkey

**Keywords:** Elderly, Social inequalities, Turkey, National, Numerical data

## Abstract

**Background:**

The objective of this study is to analyze the data of the 2018 Turkey Demographic and Health Survey and determine personal and demographic factors associated with elderly who are 60 and older and living alone.

**Methods:**

This cross-sectional study is the secondary analysis of the national data obtained with the 2018 Turkey Demographic and Health Survey. Logistic regression analysis was used to estimate differences in living alone based on gender, age, welfare status, region of residence, urban/rural residence, whether the person is working in a paid job and home ownership. Independent effect of every variable is observed in the first stage and then checked for all variables in the equation.

**Results:**

There is a total of 37,897 participants’ data in the Turkey Demographic and Health Survey Database. In the study, there are 6244 (16.5%) older adults in 11,056 households and 9.79% of the elderly population is alone. The percentage of elderly women living alone is 13.62% while this percentage is 5.48% for elderly men (*p* < 0.001). The risk of living alone for elderly women is 2.74 times more than elderly men (95% Cl 2.28–3.31). Being poor increases the risk of living alone for elderly people 2.84 fold compared to being rich (95% Cl 2.17–3.71). Those who have high school and higher education level have 2.38 (95% Cl 1.73–3.29) fold higher risk of living alone than people with lower education. Older adults living in the Western region of the country have 3.18 (95% Cl 2.20–4.59) times higher risk of living alone than older adults living in the Eastern region of the country. The risk of living alone for older adults increases 1.90 fold (95% Cl 1.55–2.32) if the house they live in do not belong to a household member.

**Conclusion:**

Based on these findings, needs of older adults under risk should be met to allow them to be healthy and live their lives in better social, economic and cultural conditions.

## Background

Old age is a period that cannot be prevented, the need for care increases with the decrease in functions, is affected by the changes in the cultural values ​​and family structure of the society, and various losses and social problems arise [1]. Today, technological developments in the field of health have led to the prolongation of life expectancy. In many countries in the world, increase in the population of people aged 60 and over is faster than other age groups. According to the World Health Organization the percentage of people over 60 years of age in the general population will increase to 22% by 2050 [2]. In Turkey, the percentage of people over the age of 65 in the population is 8.8% according to 2019 data. Similar to the general trend in the world, the number of older adults is also increasing in Turkey and the percentage of elderly population which was 8.8% in 2019 (Expected life expectancy is 75.9 years for men and 81.3 years for women) is expected to rise to 10.2% in 2023 [3]. This fast demographic transformation is typical in many developing countries which may lead to a sociological problem that these countries are unprepared for. Decrease in per capita income, depletion of resources, increased healthcare expenses are among the consequences of this situation [4, 5]. These issues cause the financial, physical and social resources available to the elderly to be insufficient. On the other hand, many older adults prefer to live alone in their own homes, with age-related issues in physical movement, regardless of their cultural background [6]. In the study of Demir et al. (2019), 9.3% of older people and in the study of Özyurt et al. (2018) conducted in Manisa, Turkey, 19.6% of older people live alone [7, 8]. Widows who have lost their husbands and represent the poorest and the most vulnerable population are also more likely to spend their older years alone [9]. Elderly people living alone are a vulnerable group that can face serious challenges to successfully ageing in place because they tend to be socially isolated, feel lonelier [6]. In the studies of Kim et al. (2019), Kim et al. (2018) and Tomiako et al. (2018) elderly people living alone have depression, less social capital, poorer health, loneliness, increased prevalence of social isolation, low social support, lower quality of life, as well as sad, hopeless and worthless showed that they were more likely to feel [10–12]. In addition, ın the study of Xu et al. (2020), 17.21% of older adults living at home were found some degree of dysfunction [13]. When living alone which is mostly combined with poverty also results in lack of healthcare, insufficient and poor nutrition, more exposure to domestic accidents as well as social isolation, lost self-confidence, ostracization, feelings of insufficiency and loneliness in the elderly [14–16]. Bingöl et al. (2010) and Parlar Kılıç et al. (2014) reported that older adults who live alone felt more functionally insufficient since they do not have any help at home [17, 18].

There are research results on the situation of elderly people living alone in Turkey [7, 8, 16, 19]. However, there is no nationwide study evaluating the personal and demographic factors associated with living alone in the elderly by examining the TDHS data, which has been systematically carried out since 1993 in order to provide data for monitoring the population and health status in Turkey. A geographical bridge connecting the East with the West, Turkey has a wide range of cultural, economic and demographic characteristics. Turkey is under the influence of both Eastern and Western cultures and the effect of these cultures vary between regions. The fact that our study examines this different sociocultural structure together and has a large sample size will fill the gap of other studies. Additionally, knowing the characteristics of elderly individuals living alone will guide both families and the public and private sectors about the issues that should be addressed as a priority.

In a country like Turkey, where older adults are under the constraints of economic and social policies, it deserves special attention to determine the differences in terms of gender, marital status, education, welfare level, geographical region, rural-urban life. Understanding socio-demographics of lonely elderly people can help to focus and provide improvement for this group which is neglected in health and social policies.

The objective of this study is to determine personal and demographic factors associated with elderly who are 60 and over living alone based on the data of 2018 Turkey Demographic and Health Survey. Our research question is “What are the personal and demographic factors associated with older people aged 60 and over living alone?”

## Methods

### Design

This cross-sectional study is the secondary analysis of the national data of the 2018 Turkey Demographic and Health Survey (TDHS).

### Participants

Regarding the dependent variable of this study, two options “living alone” and “not living alone” were used for living alone status of people over 60 years of age.

### Measurements

Independent variables were male and female for gender; no education/not finished elementary school, elementary, middle school, high school (secondary) graduate and higher education for education status. In this study secondary and higher education were combined. In TDHS data, household wealth index was determined using questions about household ownership. For wealth, groups were ranked as the richest, richer, middle, poorer, and poorest. This variable was combined in three parts (the richest, richer), middle and poor (poorer and poorest). The country was divided into five regions to analyse TDHS country data: West, South, Central, North and East and for East-West the least developed was East and the most developed was the Western region [20]. Options for place of residents were “urban” and “rural”. The options to answer the question of whether the person has an income and whether the person owns the house were yes and no. Marital status was included with three different options: married, widow and other alone (never married, divorced, never lived together). THDS is a national survey conducted every 5 years since 1993. Benefits from Turkish Statistical Institute resources, which are the national statistical institution in the standardization of variables [21].

### Data source

TDHS 2018 is a nationally representative survey which was done by Hacettepe University, Institute of Population Studies (HUIPS) as part of a global survey. This survey is repeated every 5 years. These data which focus on mother and child health are used by the Turkish Ministry of Health and many major public institutions to plan services and to allocate resources. TDHS is also the only national data which can help to have a reliable analysis of the living conditions of elderly population. Weighted, multi-stage, stratified cluster sampling was used to find the study sample. Blocks (clusters) were chosen from each stratum for primary sampling units and the total number of cluster was found to be 754. In the second stage, using the systematic random sampling method 21 households were chosen from each cluster. So the total number of households was 15.775. With 79.2% response rate, 11.056 households were accessed and 38.628 people were contacted. In line with the objective of this study, data of people who are 60 and older were analysed [22].

Two questionnaires (household and individual) were done in-person to collect data in TDHS. The household questionnaire that provided the basis for this study gives data about the size and composition of the household as well as its socio-economic status [22].

### Ethical considerations

Two thousand eighteen TDHS was reviewed and approved by the Ethics Committee of Hacettepe University. The permission to use TDHS data was obtained from the HUIPS on April 21, 2021. All methods in the study were carried out in accordance with the relevant directives and regulations.

### Data analysis

Statistical Package for the Social Sciences (v.25.0; SPSS Inc., Chicago, IL) was used for statistical analysis. Descriptive findings were shown as number and percentage distribution for categorical variables and as mean, and standard deviation for numeric variables. Logistic regression analysis was used to estimate differences in living alone according to independent variables. Regression coefficient was calculated with 95% confidence interval (95% CI) to calculate the odds ratio. Groups that have low frequency of living alone are used as the reference category. Independent effect of every variable is observed in the first stage (Crude) and then checked for all variables in the equation (Adjusted). *p* < 0.05 (two sided) was accepted as statistically significant.

## Results

There are a total of 37,897 participants in the national study (TDHS). In the study 6.244 (16.5%) people who were 60 years and older were found in 11.056 households. The mean age of the older population was 69.77 ± 8.07 and 52.91% were female. In Table 1, sociodemographic characteristics of the research population according to sex are shown. 71.07% of the older adults were married, 47.09% were elementary school graduates and 90.95% were working in a paid job and 55.54% were poor. Being widowed (39.90%), having lower education (49.98%) and working in a paid job (97.61%) were more frequently seen among women (*p* < 0.001). 9.79% of the elderly population included in the study is living alone. The percentage of elderly women living alone is 13.62% while this percentage is 5.48% for elderly men (*p* < 0.001).

**Table 1:** Distribution of socio-demographic and personal characteristics of older adults (n,%) (*n* = 6244)

Socio-demographic and personal characteristics		Total	
		n	%^**a**^
Gender	Male	2940	47.09
	Female	3304	52.91
Living alone	No	5633	90.21
	Yes	611	9.79
Marital Status (6243)	Married	4437	71.07
	Widowed	1603	25.68
	Other (Divorced, not living together)	203	3.25
Education (6184)	No Education, Preschool	2127	34.39
	Primary	2912	47.09
	Secondary and high	1145	18.52
Wealth	Poor	3468	55.54
	Middle	1150	18.42
	Rich	1626	26.04
Region	West	1998	31.99
	South	710	11.37
	Central	1310	20.98
	North	1267	20.29
	East	959	15.36
Type of place of residence	Urban	3535	56.61
	Rural	2709	43.39
Place of birth	Province center	585	9.58
	District center	1089	17.83
	Sub-istrict/village	4433	72.59
Working in a paid job (6239)	No	5662	90.75
	Yes	577	9.25
		Mean	Std. Deviation
Age	Year	69.77	8.07

^a^Percentage of the column

**Fig. 1:**
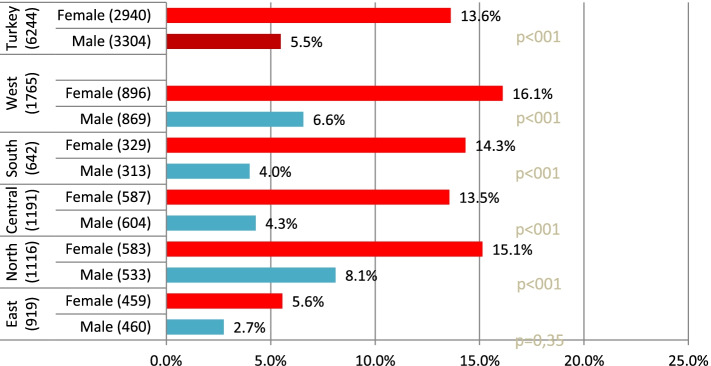
Distribution of older adults living alone in the regions according to gender (n; %)

The highest percentage of living alone is in older women living in the West with 16.1% which is followed by North (15.1%) and South (14.3%); the lowest percentage of living alone is in older men living in the East with 2.7% (Fig. 1). The percentage of older people living alone in Turkey and in all regions changes depending on the gender (*p* < 0.05).

Logistic regression analysis results for living alone are shown in Table 2. The risk of living alone in older adults was correlated with age, education level, wealth status, the region of residence and home ownership. With increasing year the risk of living alone for older adults increases by 5.4% (95% Cl 4.4–6.4). The percentage of people who are 70 years and older who live alone is 3.09 times more than those who are younger (95% Cl 2.50–3.83). The risk of living alone for elderly women is 2.74 times more than elderly men. Being poor increases the risk of living alone for elderly people 2.84 fold compared to being rich (95% Cl 2.17–3.71). Those who have no education have 2.38 (95% Cl 1.73–3.29) fold higher risk of living alone than people with high school and higher education level. Older adults living in the Western region of the country have 3.18 (95% Cl 2.20–4.59) times higher risk of living alone than older adults living in the Eastern region of the country. The risk of living alone for elderly people increases 1.90 fold (95% Cl 1.55–2.32) if the house they live in do not belong to a household member.

**Table 2:** Bivariate and multivariable logistic regression results for living alone

		n	%	Crude OR	95% Cl		***p***	Adjusted OR	95% Cl		***p***
**Gender**	Female	450	13.62	2.72	2.26	3.28	< 0.001	2.74	2.28	3.31	< 0.001
	Male (ref)	161	5.48	1.00							
Age [23].	70 and up	393	14.52	2.59	2.18	3.08		3.10	2.51	3.83	< 0.001
	< 70 (ref)	218	6.16	1.00							
Education	No Education. Preschool	234	11.00	1.43	1.11	1.85	0.006	1.44	1.17	1.78	0.001
	Primary	284	9.75	1.25	0.98	1.60	0.074	2.38	1.73	3.29	< 0.001
	Secondary and high (ref)	91	7.95	1.00	1.00						
Wealth	Poor	406	11.71	2.09	1.66	2.63	< 0.001	2.84	2.17	3.71	< 0.001
	Middle	108	9.39	1.63	1.23	2.17	0.001	1.75	1.30	2.37	< 0.001
	Rich (ref)	97	5.97	1.00							
Region	West	233	11.66	3.03	2.15	4.28	< 0.001	3.18	2.20	4.59	< 0.001
	South	68	9.58	2.43	1.63	3.64	< 0.001	2.12	1.39	3.22	< 0.001
	Central	119	9.08	2.30	1.59	3.32	< 0.001	2.66	1.81	3.91	< 0.001
	North	151	11.92	3.11	2.17	4.45	< 0.001	3.12	2.15	4.53	< 0.001
	East (ref)	40	4.17	1.00				–	–	–	
House ownership	Not	171	15.05	1.90	1.57	2.29	< 0.001	1.90	1.55	2.32	< 0.001
	Owned by a HH member (ref)	435	8.55	1.00							
Working in a paid job	No	587	10,.37	2.67	1.76	4.05	< 0.001	1,31	0.85	2.04	0.226
	Yes (ref)	24	4.16	1.00							

The risk of living alone in older women was correlated with age, education level, wealth status and the region of residence and living in a house which is owned by a household member. With increasing year, the risk of living alone in older women increases by 5.1% (95% Cl 3.9–6.2). Additionally, women who are 70 years and older have 2.92 times higher risk of living alone compared to younger women (95% Cl 2.34–3.65). Compared to uneducated poor women, those with secondary and higher education have 2.81 higher more risk of living alone (95% Cl 1.86–4.26). Poor older women have 3.16 (95% Cl 2.29–4.36) times higher risk of living alone than rich older women. In the same group living in the Western region of the country the risk of living alone increases 3.26 fold compared to living in the Eastern region (95% Cl 2.08–5.12) Those who do not own the house they live in have 2.13 (95% Cl 1.69–2.68) times higher risk than those who do (Table 3).

**Table 3:** Bivariate and multivariable logistic regression results for living alone according to gender

	**Female**										
		**n**	**%**	**Crude OR**	**95% Cl**		***p***	**Adjusted OR**	**95% Cl**		***p***
Age	70 and up	293	19.76	2.61	2.12	3.21	< 0.001	2.92	2.34	3.64	< 0.001
	< 70 (ref)	157	8.62	1							
Education	Primary	200	15.27	1.30	1.05	1.60	0.015	1.62	1.27	2.05	< 0.001
	Secondary and high	48	14.41	1.21	0.87	1.71	0.263	2.81	1.86	4.26	< 0.001
	No Education. Preschool (ref)	200	12.18	1.00							
Wealth	Poor	299	16.17	2.20	1.67	2.89	< 0.001	3.16	2.29	4.36	< 0.001
	Middle	82	13.67	1.80	1.29	2.53	0.001	2.01	1.40	2.88	< 0.001
	Rich (ref)	69	8.07	1.00				1.00			
Region	West	172	16.10	3.26	2.14	4.97	0.001	3.26	2.08	5.12	< 0.001
	South	55	14.32	2.84	1.76	4.60	0.001	2.56	1.55	4.22	< 0.001
	Central	92	13.55	2.66	1.70	4.16	0.001	3.11	1.946	4.966	< 0.001
	North	104	15.14	3.03	1.95	4.71	0.001	3.12	1.98	4.92	< 0.001
	East (ref)	27	5.56	1.00							
House ownership	Not	142	40.68	2.02	1.62	2.52	< 0.001	2.13	1.69	2.68	< 0.001
	Owned by a HH member (ref)	306	11.67	1.00							
	**Male**										
		**n**	**%**	**Crude OR**	**95% Cl**		***p***	**Adjusted OR**	**95% Cl**		***p***
Age	< 70 (ref)	61	3.55	1.00							
	70 and up	100	8..18	2..42	1..74	3.35	< 0.001	2.17	1.55	3.05	< 0.001
Wealth	Poor	107	6.61	1.88	1.23	2.87	0.004	1.90	1.22	2.96	0.005
	Rich (ref)	28	3.63	1							
Region	West	61	6.56	2.48	1.35	4.57	0.003	3.07	1.65	5.72	< 0.001
	North	47	8.10	3.12	1.67	5.84	0.001	3.30	1.75	6.21	< 0.001
	East (ref)	13	2,75	1							
Working in a paid job	No	147	6.03	2.22	1,27	3.87	0.005	1.63	0.92	2.90	0.094
	Yes	14	2.81	1							

The risk of living alone in older men is correlated with age, wealth status and the region they live in. With each increasing year, the risk of living alone in older men increases by 5.8% (95% Cl 4.0–7.7). Men who are 70 years and older have 2.17 times higher risk of living alone (95% Cl 1.55–3.05). Poor older men have 1.90 (95% Cl 1.22–2.96) fold higher risk of living alone than rich older men. In this study group, the risk of living alone is increased 3.08 (95% Cl 1.65–5.72) fold when the person is living in the Western compared to the Eastern region and 3.31 (95% Cl 1.76–6.22) compared to living in the Northern region (Table 3).

## Discussion

This study investigated the relationship between older adults living alone and socio-demographic factors and a relationship was found between the risk of living alone for older adults and gender, age, education, welfare status, region of residence and home ownership.

In the study sample one out of every ten people was alone. This finding is similar to the findings of the studies done in Cyprus (17.6%) and Hong Kong (15.9%) but quite lower than the percentages found in USA (26.0%) and EU-28 (32.1%) [24–26]. These differences and similarities with these countries can be linked to cultural and historical practices as well as economic development stages [27]. These differences provide important clues to understand and manage old age problem [28]. The increasing trend of older adults living alone is linked to less number of multi-generation families living together and the transfer of carer role from families to public institutions [28]. In addition, socioeconomic development can affect people’s preferences to live alone. As a choice, the person may want to live alone. The higher the socioeconomic status of an individual, the higher the probability of maintain good health into old age and live alone in old age [6]. Another reason is the exposure of older adults with reduced financial means to a brutal competition to have access to sources. Younger members of the family, in a harsh competition for a better live, may prefer to leave older members behind [29].

The number of older women living alone was three times the number of older men living alone. A national study conducted in Turkey in 2020 found that the percentage of older adults living alone within the elderly population was 25.04 and 75.3% of these people were women [30]. According to a study done in Iran the percentage of people living alone was 6.8 and 72.4% of those who lived alone were female [31]. According to the Statistics in the UK, there are differences in loneliness based on gender and women reported that they were more lonely, which are similar to the findings of this study [32]. In this study women who are older, poor and have higher level of education, live in the Western region and who do not have their own houses are more alone.

The risk of living alone increases three fold in women and two fold in men in the 70 years and over age group. Being married is the most common marital status in the Turkish society. However as people especially women get older, their chance of remaining married decreases. This may be caused by the fact that women tend to marry men older than themselves or life expectancy for women is longer than the life expectancy for men. That’s why women who lost their husbands spend their older years alone [9]. In Turkey, 9 out of 10 women over the age of 90 have lost their husbands [33]. The number of older women whose spouses are dead is 4 times higher than the number of older men whose spouses are dead [34]. This can be the reason why more older women live alone.

Our study found that the number of older men is higher than the number of older women in all education levels. The education level of women is significantly lower. In addition to these the study shows that education level does not affect the state of being alone for men whereas the number of women with higher education levels living alone is almost three times more than women with lesser education. Women with higher education are known to get married later and lower number of women with higher education are getting married [35]. This may explain the reason why educated women tend to live alone more.

In this study low level of wealth increases the risk of living along for older adults almost three fold and the risk of living alone increases with increasing poverty. Losing a spouse at old age does not only lead to loneliness but also reduces income. More than half of one person households of older adults are also in the lowest income group. Considering that most of the older adults living alone are women and poverty is the most important problem in one person households, it is possible to conclude that the most vulnerable population group in Turkey for poverty and income are older women [9, 36–38]. Additionally, similar to the findings of our study, these studies show that poor women are more lonely. Another parameter that stood out in this study is whether the family has the ownership of the house. This factor which is a wealth indicator is found to be related with living alone for older women. Family fortune is reported to be one of the determinants of living alone at old age [39]. As a result, loneliness in old age may be an outcome of women’s poverty and lack of education, as well as a choice of educated women with socioeconomic freedom.

In this study, the highest percentage of living alone is in older women living in the West and in the North with 16.1% while the lowest percentage of living alone is in older men living in the East with 2.7%. In this study there are 3 times more older people living the Western region, which is the most developed region of Turkey than Eastern region. The percentage of older people living alone in Turkey and in all regions changes significantly depending on the gender. Eastern Turkey is the least developed region of the country and has the lowest per capita income [40]. Due to internal migration from Eastern Turkey to more developed regions of the country, population increase rate is low and maternal and infant mortality rates are high in this region On the contrary, Western Turkey is the most developed region and has a higher level of demographic transformation [41]. Differences in regions in our study can be explained with these regions being in different stages of development. Furthermore, in addition to the fact that regions are in different stages of economic development, cultural differences might also have caused this difference. Cultural practices, increasing trend of living alone in Western parts of the country favour small families or single parent households. On the other hand, patriarchal, male-dominant, extended family structure is still preferred in the East [42, 43]. Furthermore these cultural differences may explain why older women are more lonely than older men in the western parts of the country.

### Limitations of the study

This study evaluated gender, education level, marital status, welfare level and region of residence of older adults who live alone based on national data however health status and functionality of these people were not evaluated. Studies that investigate the trend of living alone among older adults have different methodologies; some studies evaluate households whereas others evaluate individuals. Therefore evaluations of individuals in this study can make it difficult to make comparisons with other studies.

## Conclusions

This study, it can be concluded that the risk of living alone in older adults is higher in women, in poor people, in people who live in a house that is not owned by themselves or by their families, in people who have higher level of education and in people who live in the Western part of the country. When considered as a whole older adults who are struggling to be present within the context of old age, poverty and loneliness can have difficulties in meeting their basic needs and having access to sufficient care. Based on the striking findings of our study, healthcare services for older adults, institutional care services, social security policies should be planned to be comprehensive and holistic taking into consideration gender, loneliness, region of residence, poverty and education level of older adults.

## Data Availability

The permission to use TDHS data was obtained from the HUIPS. The datasets that support the findings of this study are available from the HUIPS upon reasonable request.

## Abbreviations

TSI: Turkish Statistical Institute
WHO: World Health Organization
THDS: Turkey Demographic and Health Survey
HUIPS: Hacettepe University, Institute of Population Studies
